# Can patient-led surveillance detect subsequent new primary or recurrent melanomas and reduce the need for routinely scheduled follow-up? A protocol for the MEL-SELF randomised controlled trial

**DOI:** 10.1186/s13063-021-05231-7

**Published:** 2021-05-04

**Authors:** Deonna M. Ackermann, Amelia K. Smit, Monika Janda, Cathelijne H. van Kemenade, Mbathio Dieng, Rachael L. Morton, Robin M. Turner, Anne E. Cust, Les Irwig, Jolyn K. Hersch, Pascale Guitera, H. Peter Soyer, Victoria Mar, Robyn P. M. Saw, Donald Low, Cynthia Low, Dorothy Drabarek, David Espinoza, Jon Emery, Peter Murchie, John F. Thompson, Richard A. Scolyer, Anthony Azzi, Alister Lilleyman, Katy J. L. Bell

**Affiliations:** 1 Sydney School of Public Health, Faculty of Medicine and Health, The University of Sydney, Sydney, Australia; 2 Cancer Epidemiology and Prevention Research, Sydney School of Public Health, Faculty of Medicine and Health, The University of Sydney, Sydney, Australia; 3 Melanoma Institute Australia, The University of Sydney, Sydney, Australia; 4 Centre for Health Services Research, The University of Queensland, Brisbane, Australia; 5 NHMRC Clinical Trials Centre, The University of Sydney, Sydney, Australia; 6 Biostatistics Centre, University of Otago, Dunedin, New Zealand; 7 Faculty of Medicine and Health, The University of Sydney, Sydney, Australia; 8 Sydney Melanoma Diagnostic Centre, Royal Prince Alfred Hospital, Sydney, Australia; 9 Dermatology Research Centre, The University of Queensland Diamantina Institute, The University of Queensland, Brisbane, Australia; 10 Department of Dermatology, Princess Alexandra Hospital, Brisbane, Australia; 11 Victorian Melanoma Service, Alfred Health, Melbourne, Australia; 12 School of Public Health and Preventive Medicine, Monash University, Melbourne, Australia; 13 Division of Surgery, Royal Prince Alfred Hospital, Sydney, Australia; 14 Cancer Voices NSW, Sydney, Australia; 15 Centre for Cancer Research, Faculty of Medicine, Dentistry and Health Sciences, University of Melbourne, Melbourne, Australia; 16 Institute of Applied Health Sciences, University of Aberdeen, Aberdeen, United Kingdom; 17 Tissue Pathology and Diagnostic Oncology, Royal Prince Alfred Hospital and NSW Health Pathology, Sydney, Australia; 18 Newcastle Skin Check, Newcastle, Australia; 19 Faculty of Medicine, University of Queensland, Brisbane, Australia

**Keywords:** Melanoma, Cancer surveillance, Early detection of cancer, Self-examination, Teledermoscopy, Telehealth, Randomised controlled trial, Health services research

## Abstract

**Background:**

Most subsequent new primary or recurrent melanomas might be self-detected if patients are trained to systematically self-examine their skin and have access to timely medical review (patient-led surveillance). Routinely scheduled clinic visits (clinician-led surveillance) is resource-intensive and has not been shown to improve health outcomes; fewer visits may be possible if patient-led surveillance is shown to be safe and effective. The MEL-SELF trial is a randomised controlled trial comparing patient-led surveillance with clinician-led surveillance in people who have been previously treated for localised melanoma.

**Methods:**

Stage 0/I/II melanoma patients (*n* = 600) from dermatology, surgical, or general practice clinics in NSW Australia, will be randomised (1:1) to the intervention (patient-led surveillance, *n* = 300) or control (usual care, *n* = 300). Patients in the intervention will undergo a second randomisation 1:1 to polarised (*n* = 150) or non-polarised (*n* = 150) dermatoscope. Patient-led surveillance comprises an educational booklet, skin self-examination (SSE) instructional videos; 3-monthly email/SMS reminders to perform SSE; patient-performed dermoscopy with teledermatologist feedback; clinical review of positive teledermoscopy through fast-tracked unscheduled clinic visits; and routinely scheduled clinic visits following each clinician’s usual practice. Clinician-led surveillance comprises an educational booklet and routinely scheduled clinic visits following each clinician’s usual practice.

The primary outcome, measured at 12 months, is the proportion of participants diagnosed with a subsequent new primary or recurrent melanoma at an unscheduled clinic visit. Secondary outcomes include time from randomisation to diagnosis (of a subsequent new primary or recurrent melanoma and of a new keratinocyte cancer), clinicopathological characteristics of subsequent new primary or recurrent melanomas (including AJCC stage), psychological outcomes, and healthcare use. A nested qualitative study will include interviews with patients and clinicians, and a costing study we will compare costs from a societal perspective. We will compare the technical performance of two different models of dermatoscope (polarised vs non-polarised).

**Discussion:**

The findings from this study may inform guidance on evidence-based follow-up care, that maximises early detection of subsequent new primary or recurrent melanoma and patient wellbeing, while minimising costs to patients, health systems, and society.

**Trial registration:**

Australian New Zealand Clinical Trials Registry (ANZCTR): ACTRN12621000176864. Registered on 18 February 2021.

**Supplementary Information:**

The online version contains supplementary material available at 10.1186/s13063-021-05231-7.

## Administrative information

The order of the items has been modified to group similar items (see http://www.equator-network.org/reporting-guidelines/spirit-2013-statement-defining-standard-protocol-items-for-clinical-trials/).

**Table:** 

Title {1}	Can patient-led surveillance detect subsequent new primary or recurrent melanoma and reduce the need for routinely scheduled follow-up? A protocol for the MEL-SELF randomised controlled trial. Trial Acronym: MEL-SELF (MELanoma SELF-surveillance)
Trial registration {2a and 2b}.	Australian New Zealand Clinical Trials Registry: ACTRN12621000176864
Protocol version {3}	Version 1, 19 February 2021.
Funding {4}	National Health & Medical Research Council (NHMRC) Project grant (APP1163054).
Author details {5a}	Deonna M. Ackermann,^1X^ Amelia K Smit,^2,3,X^ Monika Janda,^4^ Cathelijne H. van Kemenade,^1^ Mbathio Dieng,^5^ Rachael L Morton,^3,5^ Robin M Turner,^6^ Anne E Cust,^2,3^ Les Irwig,^1^ Jolyn k. Hersch,^1^ Pascale Guitera,^3,7,8^ H. Peter Soyer,^9,10^ Victoria Mar,^11,12^ Robyn PM Saw,^3,7,13^ Donald Low,^14^ Cynthia Low,^14^ Dorothy Drabarek,^1^ David Espinoza^5^, Jon Emery,^15^ Peter Murchie,^16^ John F. Thompson,^3,7,13^ Richard A. Scolyer,^3,7,17^ Anthony Azzi,^18,19^ Alister Lilleyman,^18,19^ Katy J.L. Bell^1^ ^X^DA and AKS contributed equally to this paper. ^1^ Sydney School of Public Health, Faculty of Medicine and Health, The University of Sydney, Sydney, Australia ^2^ Cancer Epidemiology and Prevention Research, Sydney School of Public Health, Faculty of Medicine and Health, The University of Sydney, Sydney, Australia ^3^ Melanoma Institute Australia, The University of Sydney, Sydney, Australia ^4^ Centre for Health Services Research, The University of Queensland, Brisbane, Australia ^5^ NHMRC Clinical Trials Centre, The University of Sydney, Sydney, Australia ^6^ Biostatistics Centre, University of Otago, Dunedin, New Zealand ^7^ Faculty of Medicine and Health, The University of Sydney, Sydney, Australia ^8^ Sydney Melanoma Diagnostic Centre, Royal Prince Alfred Hospital, Sydney, Australia ^9^ Dermatology Research Centre, The University of Queensland Diamantina Institute, The University of Queensland, Brisbane, Australia ^10^ Department of Dermatology, Princess Alexandra Hospital, Brisbane, Australia ^11^ Victorian Melanoma Service, Alfred Health, Melbourne, Australia ^12^ School of Public Health and Preventive Medicine, Monash University, Melbourne, Australia ^13^ Division of Surgery, Royal Prince Alfred Hospital, Sydney, Australia ^14^ Cancer Voices NSW, Sydney, Australia ^15^ Centre for Cancer Research, Faculty of Medicine, Dentistry and Health Sciences, University of Melbourne, Melbourne, Australia ^16^ Institute of Applied Health Sciences, University of Aberdeen, Aberdeen, United Kingdom ^17^ Tissue Pathology and Diagnostic Oncology, Royal Prince Alfred Hospital and NSW Health Pathology, Sydney, Australia ^18^ Newcastle Skin Check, Newcastle, Australia ^19^ Faculty of Medicine, University of Queensland, Brisbane, Australia
Name and contact information for the trial sponsor {5b}	Sponsor: The University of Sydney. Contact name: Associate Professor Katy Bell Address: Sydney School of Public Health, Edward Ford Building (A27), The University of Sydney NSW 2006. Phone: +612 93514823 Email: katy.bell@sydney.edu.au
Role of sponsor {5c}	Sponsors and funders did not have any role in study design; collection, management, analysis or interpretation of data; writing of the report; or the decision to submit the report for publication and do not have ultimate authority over any of these activities.

## Introduction

### Background and rationale {6a}

Melanoma incidence continues to increase in many countries worldwide. In Australia, the total number of people diagnosed with new melanomas increased from 3526 in 1982 to 14,485 in 2016, and the age-standardised rate increased from 27.9 per 100,000 in 1982 to 54 per 100,000 in 2016 [1]. This appears to be largely driven by increased early detection of early-stage melanoma before it has spread from the skin (in situ (stage 0) melanoma, and stage I–II invasive melanoma, together > 95% of all new melanoma diagnoses in Australia) [2]. After surgical excision of the melanoma, these patients are at risk of developing a subsequent new primary melanoma, a recurrence of their treated primary melanoma, and new keratinocyte (non-melanoma) skin cancers. Therefore, it is typically recommended that they undergo at least 10 years follow-up (and often longer), at intervals ranging from 3 to 12 months depending on melanoma stage [3, 4]. Patients diagnosed with an early-stage melanoma have a very good prognosis in terms of life expectancy: those with melanoma in situ (stage 0) have the same mortality risk as the general population, and those with thin melanomas (< 0.8 mm, which accounts for 65% of all invasive melanomas) have a 20-year survival of 80–96% [5–7]. Clinician-led surveillance in the form of routinely scheduled clinic visits is widely accepted as the usual model of follow up care after removal of a melanoma, under the assumption that this leads to earlier detection and treatment a subsequent new primary or recurrent melanoma, and reduced mortality. However, there is no direct evidence that clinician-led surveillance leads to improved survival [4, 8].

There is a need to balance the potential benefits of clinician surveillance for a subsequent new primary or recurrent melanoma, and keratinocyte skin cancers, against costs and possible psycho-social harms of frequent routinely scheduled clinic visits and investigations [9, 10]. The costs of follow-up for early-stage melanoma are substantial and have been estimated at AU$44 M over 5 years for American Joint Committee of Cancer (AJCC7) stage I/II [11]. Routinely scheduled clinic visits also cause opportunity costs in terms of clinician and patient time and could cause delays in the assessment of new patients who might benefit from more timely evaluation and treatment. Patients also incur travel and parking expenses, which may be especially burdensome for those who do not live close to the treatment centre. Fewer routinely scheduled clinic visits may have little impact on the detection of subsequent new primary or recurrent melanomas [12] and could result in substantial cost savings [13–15].

In addition to these considerations, the ongoing climate emergency and novel coronavirus (COVID-19) pandemic are both contributing to a growing and urgent need to re-consider how healthcare service is delivered [16, 17]. Other models of follow-up, including those using telehealth, present an opportunity to potentially decrease low value or unnecessary care [18], reduce environmental impacts of healthcare [17], and reduce nosocomial infection risk [19]. Telehealth may be especially valuable in the Australian context where a up to 30% of the population live remote to the major cities [20].

Patient-led surveillance is a new model of follow-up care for patients with a history of early-stage melanoma. Compared to the traditional clinician-led approach, there is increased reliance on patient self-management of their melanoma risk, with increased support for skin self-examination (SSE), fast-tracked access to unscheduled clinic visits should the patient identify a concerning lesion, and the potential for fewer routinely scheduled clinic visits [21]. Self or partner-detection of abnormalities may allow early detection of a subsequent new primary or recurrent melanoma, which may increase the effectiveness of treatment, and improve survival [22–25]. Although SSE is universally recommended by clinical guidelines, SSE education and practice remain suboptimal [21, 26]. Studies to date suggest that few people carry out SSE thoroughly and many fail to complete important components such as checking hard-to-see locations like the scalp, seeking assistance from someone else, and documenting changing lesions [27]. Previous survey and interview studies with people undergoing melanoma follow-up have shown a need for increased support for SSE, and acceptance of decreased frequency of routinely scheduled follow-up if recommended by their clinician [21, 28]. Additionally, clinicians are more likely to recommend fewer routinely scheduled clinic visits if they are confident of the patient’s ability to conduct SSE [29].

Digital technologies offer accessible platforms to facilitate SSE support and consolidate the behaviour changes needed for patient-led surveillance. Smartphone and web-based applications (apps) can prompt, remind, instruct, and record results from SSE, and facilitate teledermoscopy through the transmission of digital images taken by the patient, to a dermatologist for review. The Achieving Self-directed Integrated Cancer Aftercare (ASICA) Skin Checker app has been found to be feasible and acceptable for supporting SSE and triaging clinical review [30]. Studies on the preliminary assessment of diagnostic accuracy of patient-performed mobile teledermoscopy have demonstrated feasibility and acceptability for skin surveillance [31, 32]. Our pilot randomised controlled trial in patients with early-stage melanoma (*n* = 100) demonstrated the feasibility of a patient-led surveillance intervention that incorporated both the ASICA app and patient-performed mobile teledermoscopy (manuscript in preparation) [33]. This intervention also appeared to improve knowledge, attitudes, and practice of SSE, and to increase the early detection of subsequent new primary melanomas, with no adverse psychological outcomes. A larger sample is needed to confirm or refute these preliminary findings on the effects of patient-led surveillance.

To address these evidence gaps, we will conduct the MELanoma SELF surveillance (MEL-SELF) study. This article presents the protocol for a randomised control trial that aims to test the hypotheses that patient-led surveillance results in better health and psychological outcomes than clinician-led surveillance and consumes fewer healthcare resources (if implemented as a replacement rather than an add on to routinely scheduled clinic visits).

### Objectives {7}

#### Primary objective

To assess whether patient-led surveillance (comprising: smartphone supported SSE, teledermatology, fast-tracked unscheduled clinic visits in addition to routinely scheduled clinic visits) compared to clinician-led surveillance (usual care using treating doctor’s usual processes for fast-tracked unscheduled and for routinely scheduled clinic visits) increases the proportion of participants who are diagnosed with a subsequent new primary or recurrent melanoma at a fast-tracked unscheduled clinic visit.

#### Secondary objectives

To assess the impact of patient-led vs clinician-led surveillance on:

1. Time from randomisation to diagnosis of a subsequent new primary or recurrent melanoma and of a keratinocyte cancer.
2. Psychological outcomes using validated scales for fear of new or recurrent melanoma, general anxiety, stress, and depression.
3. Skin self-examination:

I. confidence in, knowledge of, attitudes to, and beliefs about skin self-examination;
II. adherence to recommended skin self-examination practice.

4. Acceptability of a reduction in routinely scheduled clinic visit frequency.
5. Resource use through measurement of:

I. number of lesions surgically excised;
II. number of follow-up visits attended (both scheduled and unscheduled).

And to:

6. Evaluate the technical performance of two different models of mobile dermatoscope, which patients can attach to their smartphones.
7. Assess the acceptability of patient-led surveillance to patients and clinicians (nested qualitative study).
8. Assess resource use and costs for healthcare system, patient, and environment (costing study).

### Trial design {8}

**Fig. 1:**
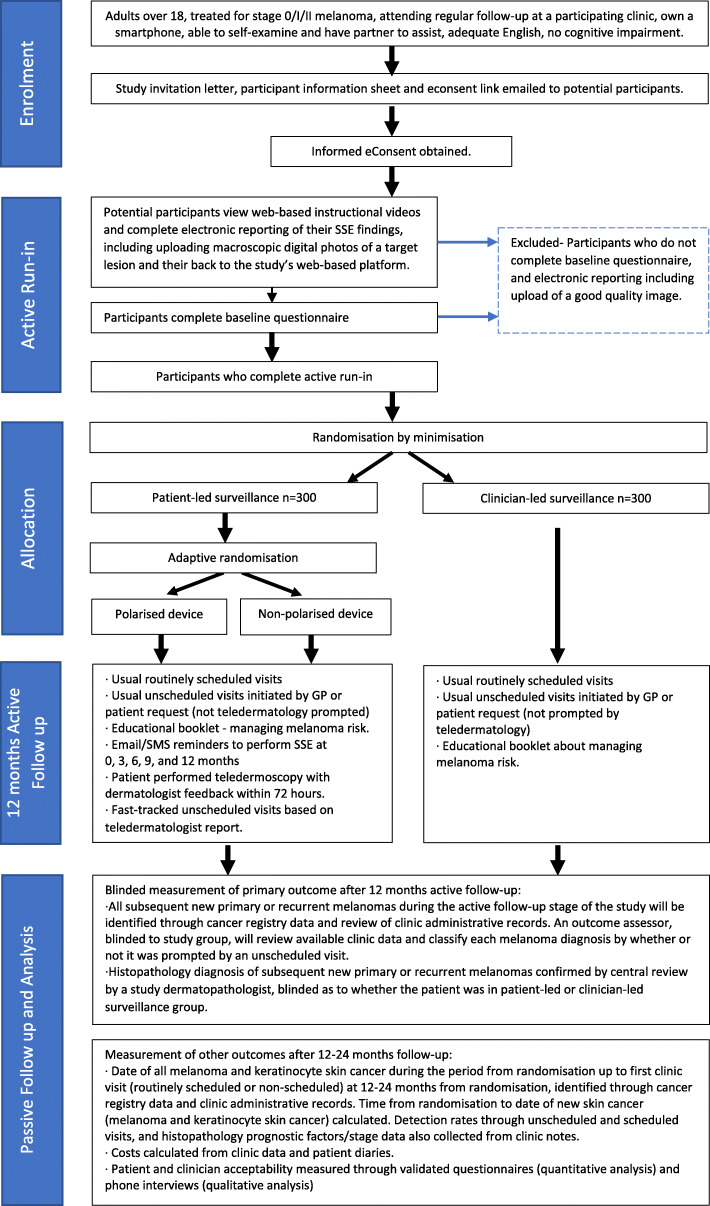
Study flow diagram

This two-arm, parallel, superiority randomised controlled trial will recruit 600 participants with a 1:1 allocation ratio between intervention and control groups (see Fig. 1 for trial flow diagram). A second randomisation step within the intervention arm will randomise participants 1:1 into alternative models of mobile dermatoscopes (polarised or non-polarised light source). This will allow robust evaluation of the technical performance of models with differing price points that represent the range of technologies currently on the market [34]. The primary outcome and secondary outcomes for the intervention group (both models of dermatoscope combined) will be compared to the control group.

#### Costing study

This will estimate costs to the health care system and to patients for the intervention and control groups. All costs associated with skin self-examination, follow-up clinics, management of newly identified lesions, and any other out of pocket costs will be included. We will provide total costs, and disaggregated costs for different types of activity within the intervention and control groups (e.g. separate cost estimates for fast-tracked unscheduled clinic visits and for routinely scheduled clinic visits). We will also measure and value all resources, and estimate the carbon emissions associated with melanoma surveillance activities in the intervention and control groups using established methods for carbon costing [35, 36]. Further details will be available in the trial’s costing study protocol and health economic analysis plan (manuscript in preparation).

#### Nested qualitative study

A nested qualitative study will explore patients’ and clinicians’ satisfaction with the intervention and its acceptability and explore components which may need to be changed for implementation into routine clinical practice (see section 20a and Supplementary file 3).

## Methods: participants, interventions, and outcomes

### Eligibility criteria {10}

#### Inclusion criteria

Patients who:

- Have completed treatment for early-stage (defined as stages: in situ/I/II) melanoma and are attending regular melanoma follow-up as indicated by at least one routinely scheduled visit booked within the next 12 months at a recruiting treatment centre;
- Are able to conduct SSE;
- Have a suitable study partner (spouse, partner, family member, friend) to help with SSE;
- Own a smartphone (and have access to internet, email, and SMS text messaging)
- Routinely scheduled clinic visit frequency at the treatment centre is 6 monthly or less frequent
- Are able to give informed consent
- Has sufficient English language skills to read the materials and complete the questionnaires
- Are at least 18 years of age

#### Exclusion criteria

Patients who:

- Have ever had stage III/IV melanoma.
- Have a known past or current diagnosis of cognitive impairment.
- Participated in the MEL-SELF pilot trial (conducted Nov 2018–Feb 2020) [33].
- Own a smartphone that is not compatible with the mobile dermatoscopes that are part of the intervention.

### Study setting {9}

Individuals who meet the eligibility requirements will be recruited from three sites in New South Wales (NSW), Australia. These are at the Royal Prince Alfred Hospital and the Melanoma Institute Australia (North Sydney), which are specialist-led clinics in metropolitan Sydney, and the Newcastle Skin Check clinic, which is a primary care skin cancer clinic run by general practitioners located in metropolitan Newcastle. Further sites may be opened if needed to meet the recruitment target and may include regional clinics.

### Who will take informed consent? {26a}

Participating clinicians will identify patients attending their clinic who are eligible for the trial. During this consultation, clinicians will discuss the research study with the patient, answer any questions, and identify a target lesion which the participant will monitor during the study (the most concerning or remarkable lesion that the treating doctor would like to monitor for changes). Following this visit, research study staff will email the patient with an invitation package comprising an invitation letter, participant information sheet and a link to an online consent form (eConsent will be provided in an online data collection platform, REDCap [37, 38] hosted by The University of Sydney). Potential participants will be informed that participation is voluntary and that their decision about participating will have no impact on their clinical care. Research study staff will telephone patients who do not complete their consent form within 2 weeks. If the patient does not return their consent form after this telephone call, no further contact will be made, and this information will be recorded.

#### Active run-in phase

Once participants provide eConsent, research study staff will email participants with instructions to:

- complete an online baseline questionnaire;
- log-in to the web-based ASICA skin checker platform, view instructional videos, and undertake guided total skin self-examination and electronic reporting of their findings;
- upload a macroscopic digital photo using their smartphone of the predetermined target lesion (chosen by the treating clinician) and a photo of their back (to document the amount of sun damage to provide dermatologists with an indicator of melanoma risk) to REDCap.

Eligible patients who consent to participate and complete all the run-in activities within 2 months of consenting, including submission of photos of sufficient quality to allow dermatological assessment, will be randomised.

### Additional consent provisions for collection and use of participant data and biological specimens {26b}

On the eConsent form, participants will be asked for permission for their doctors, other health professionals, hospitals or laboratories, the NSW Cancer Registry, to release information to The University of Sydney concerning their disease and treatment for the purposes of this project. Specific consent for linkage to Medicare claims data will be sought using the Services Australia approved template. Participants will be asked to consent to the storage and use of their skin images during the research project and for future use. Future use may involve further comparison of the performance of the two dermatoscope devices, and comparison of teledermatology reports across different dermatologists. This trial does not involve collecting biological specimens for storage.

### Interventions

#### Explanation for the choice of comparators {6b}

Usual care for early-stage melanoma patients in Australia comprises routinely scheduled visits as recommended by treating clinicians, with the possibility of scheduling additional visits if needed, and information pamphlets on melanoma early detection; therefore, these conditions will be applied to the control arm. The intervention arm will include all the same conditions as the control arm, in addition to teledermatology supported SSE—this is a potential new approach to patient follow-up.

##### Intervention description {11a}

Trial participants in the control arm will receive clinician-led surveillance (usual care), which involves:

- An educational booklet ‘Your guide to early melanoma’;
- Routinely scheduled clinic visits as recommended by their treating clinician (likely to be at intervals between 6 and 12 months);
- Unscheduled clinic visits, if needed (not prompted through teledermatology)

**Fig. 2:**
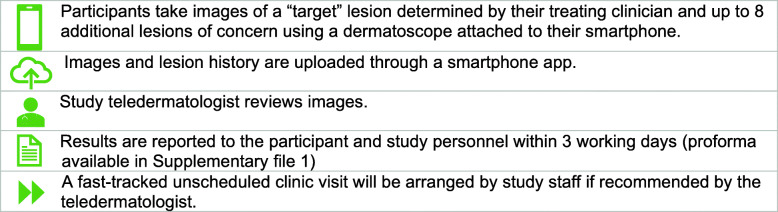
Teledermatology process

Trial participants in the intervention arm will receive the intervention (patient-led surveillance) as an adjunct to their usual care, including:

- An educational booklet ‘Your guide to early melanoma’;
- Routinely scheduled visits as recommended by their treating clinician;
- Unscheduled clinic visits, if needed (not prompted through teledermatology);
- Continued access to the ASICA skin checker instructional videos on how to perform SSE (including checking for locoregional recurrence) [30, 39];
- A mobile dermatoscope (with a polarised or non-polarised light source) to attach to their phone, with detailed written and video instructions on how to use the smartphone app and dermatoscope
- Training for themselves and a partner/friend/support person in using the dermatoscope and app, delivered one-on-one by web conferencing;
- Email or SMS text reminders every 3 months to perform SSE;
- Teledermatology (see Fig. 2)

### Criteria for discontinuing or modifying allocated interventions {11b}

The assigned study intervention may need to be modified or discontinued by trial investigators for various reasons including:

- Harms and safety issues as detailed in sections 22 and 30.
- Participant nonadherence. Less frequent submission of images could be offered to participants if necessary.

Study participants will be retained in the trial whenever possible to enable follow-up and measurement of primary and secondary outcomes.

### Strategies to improve adherence to interventions {11c}

Strategies to improve participant adherence to the intervention include:

- Participants will receive an overall schedule of trial-related tasks, and automated reminders delivered via text messages (SMS) and emails. These will be supplemented by additional SMS, email, and phone calls from research study staff, if tasks are overdue.
- There will be detailed initial training and participants will have ongoing access to written and video instructions on the use of the smartphone app and dermatoscope.
- Participants will have direct access to research study staff via text messaging with phone calls and web-conferencing arranged as needed.

We will report the number and proportion of participants adhering to three-monthly image submission (intervention group only) and using non-trial melanoma surveillance including telehealth and other imaging tests for the skin (intervention and control groups).

### Relevant concomitant care permitted or prohibited during the trial {11d}

There are publicly available apps and other forms of telehealth that assess the risk of melanoma in pigmented lesions using a smartphone camera and automated analysis. Recent systematic reviews have found that these were not of sufficient quality to be recommended, as the apps have low sensitivity in detecting melanoma and may give false reassurance to patients [40, 41]. Current clinical practice guidelines recommend against the use of publicly available melanoma apps. The participant information sheet advises patients that these apps are not recommended. We will measure participant use of non-trial technologies for melanoma detection by questionnaire at 6 and 12 months and will adjust for this at the analysis stage.

### Provisions for post-trial care {30}

All participants will return to usual care after the trial. As part of our safety protocol, we will monitor the self-reported questionnaires measuring depression, anxiety, and stress (DASS-21). Any participants with scores above the “severe” threshold will be contacted by the site coordinator who will indicate concern, ask if they would like help/support, and offer to facilitate this through contact with the treatment team who would manage referrals (the thresholds we will use are: depression subscale ≥ 11, anxiety subscale ≥ 8, and stress subscale ≥ 13).

### Outcomes {12}

#### Primary outcome

(M1) Proportion of participants who are diagnosed with a subsequent new primary or recurrent melanoma (any stage) at a fast-tracked unscheduled clinic visit during the 12 months follow-up after randomisation Melanomas are histologically confirmed and centrally reviewed by the trial dermatopathologist (RAS), who will be blinded to study arm. Classification of a visit as fast-tracked unscheduled vs routinely scheduled will be done by the endpoint adjudication committee based on the participant’s clinic letters, blinded to study arm.

#### Secondary outcomes

(M2) Time to diagnosis of a new skin cancer: time from randomisation to the histopathology diagnosis of a melanoma or keratinocyte skin cancer (as defined by the date on the histopathology report).

(M3) Pathological characteristics of new skin cancers: including thickness, stage, and other prognostic factors (melanomas and keratinocyte skin cancers).

(M4) Skin Self Examination (SSE) including:

M4.1. Thoroughness, confidence, beliefs, attitude, and knowledge of SSE will be assessed by items adapted from Janda et al. [42].

M4.2. Adherence with recommended SSE practice (total body self-examination conducted three-monthly); Participants will be asked how often they perform a complete examination of their skin.

(M5) Fear of new or recurrent melanoma (FCR) severity: assessed using a modified (i.e. melanoma-specific) version of the 9 item FCR Inventory severity subscale, the most comprehensive multi-dimensional scale of FCR available [10]. A higher score indicates greater FCR.

(M6) General anxiety, stress and depression: measured using the short version of the Depression Anxiety and Stress Scales (DASS-21) [43]. The DASS-21 is a set of three 7-item self-report scales designed to measure the emotional states of depression, anxiety, and stress.

(M7) Acceptability of hypothetical reduction in scheduled clinic visit frequency: measured through questionnaire items designed specifically for this study.

(M8) Number of lesions surgically evaluated: measured through interrogation of clinic data.

(M9) Number of clinic visits attended: (routinely scheduled and fast-tracked unscheduled clinic visits) measured through clinic data.

(M10) Resource use: Costs to the health system and to patients in each arm of the trial will be estimated using an online resource use diary. The diary will be used to document and measure health service use - such as doctors’ clinic visits, other health practitioner consultations, imaging and other tests, hospital visits, and time taken to take and submit images for teledermatology. The diary will also document days out-of-role (including paid and unpaid work) and travel costs. The diary will be based on existing resource use questionnaires and the patient diary used in the MEL-SELF pilot study.

(M11) Carbon emissions: carbon costs of resources used, estimated using established carbon accounting methods [35, 36].

(M12) Qualitative results: telephone interviews with a sub-set of intervention group participants at baseline, 6 months and 12 months after randomisation and control group participants at 12 months to explore, in depth, patients’ experiences from participation in the trial. We will also interview clinicians to explore their experience of the benefits and limitations of the intervention. A protocol for the nested qualitative study is included in Supplementary file 3.

(M13) Technical performance of dermatoscopes: participant ability to submit images (adherence with 3 monthly image submission), participant satisfaction with dermatoscope, quality of the images (measured using a checklist developed and tested in another teledermatology study [44]), any device deficiencies reported.

### Participant timeline {13}

**Fig. 3:**
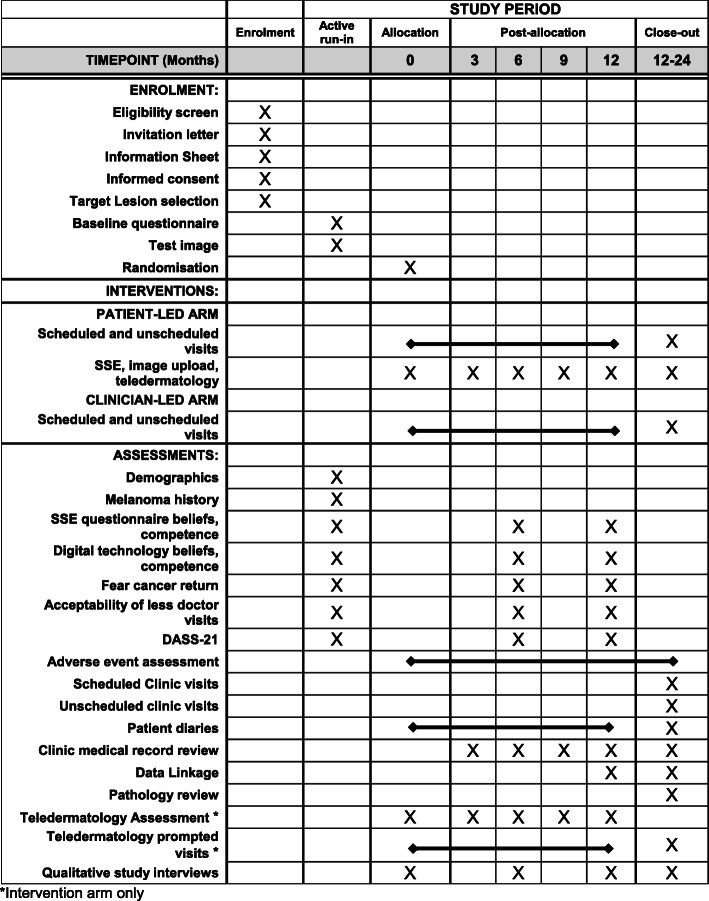
Participant schedule

The components and timing of enrolment activities, interventions, and assessments for participants are shown in Fig. 3. Follow up will continue for 12 months. Participants in the intervention group will perform SSE and submit images at baseline (post-randomisation), 3, 6, 9, and 12 months post randomisation. Outcomes will be assessed by participant-reported questionnaires at baseline (pre-randomisation), 6 and 12 months after randomisation.

### Sample size {14}

Assuming that 6–8% of patients in the clinician-led surveillance group have a subsequent new primary or recurrent melanoma diagnosed within the 12 months follow up [21, 45] and that 1% have a diagnosis through a fast-tracked unscheduled clinic visit, we will need to recruit at least 452 participants (226 to patient-led surveillance and 226 to clinician-led surveillance) in order to have at least 80% power to detect a 5% absolute increase in the patient-led surveillance group (i.e. 6% have new or recurrent melanoma diagnosed through unscheduled visit at treatment centre). Assuming up to 25% of study participants withdraw consent or dropout, we will recruit 600 participants (300 to patient-led surveillance and 300 to clinician-led surveillance). These calculations assume a two-sided 5% significance level and were done using Fisher’s exact test. This sample size will also ensure at least 80% power to detect a hazard ratio of 1.71 for time from randomisation to diagnosis of a skin cancer for the patient-led vs clinician-led surveillance groups (due to earlier and increased detection in the patient-led group). This calculation assumes a 20% event rate in the clinician-led surveillance group [21, 45] (60 events among 300 control participants), a 32% event rate in the patient-led surveillance group (96 events among 300 intervention participants), and 26% event rate overall (156 events among 600 trial participants).

For the nested qualitative study, we will aim to recruit 30–45 participants, which may vary depending on the saturation of themes but is a sample size commonly sufficient to reach saturation in themes and topics [46].

### Recruitment {15}

Strategies for achieving adequate participant enrolment include training and support for clinical staff at each site, provision of one-page flyers for patients in clinic waiting rooms, provision of one-page summary of the trial tasks for recruiting clinicians, and regular monitoring of recruitment targets. Administrative data from the Melanoma Institute Australia (MIA), indicates that approximately 800–900 new patients are treated for early melanoma at MIA each year and approximately 50% then attend regular follow-up at MIA (includes MIA clinics in North Sydney and Royal Prince Alfred Hospital clinics) [47]. The pilot trial found that 21% of people screened were randomised [33]. Thus, recruiting 600 patients over 2 years from all patients with a history of early melanoma who are attending follow-up (new and existing patients) at MIA, Royal Prince Alfred Hospital or the GP-run Newcastle Skin Check clinics appears feasible.

### Assignment of interventions: allocation

#### Sequence generation {16a}

Participants will be randomly assigned to either control or intervention arms with a 1:1 allocation ratio. Minimisation will be used to ensure the study groups are balanced on key prognostic factors:

- Specialist versus GP led clinic (two specialist clinic sites = Melanoma Institute Australia/Royal Prince Alfred Hospital and one GP led site Newcastle Skin Check)
- Patient age (age groups = 18–39, 40–70, 71+)
- Gender (male, female, other)
- Melanoma stage (Stage 0, IA, IB, IIA, IIB, IIC)
- Risk of a subsequent new primary melanoma (1-year absolute risk score < 5%, 5–10%, > 10% [48]).
- Dysplastic naevus syndrome (yes or no).

**Fig. 4:**
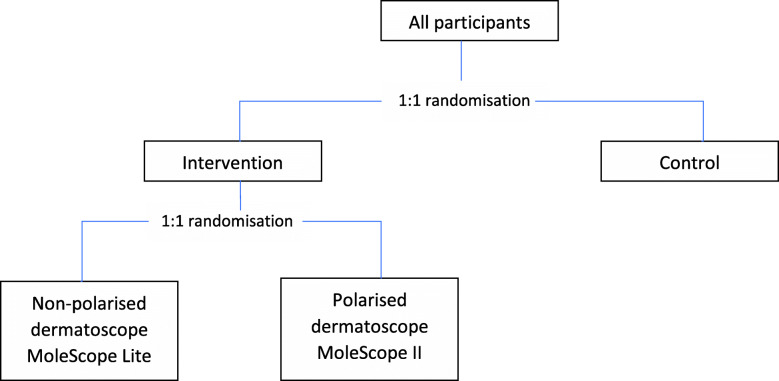
Two-stage randomisation in MEL-SELF trial. *Polarised device refers to cross-polarised light dermatoscope, and non-polarised device refers to natural light dermatoscope

Within the intervention arm, participants will be randomised 1:1 to one of the two models of mobile dermatoscope (see Fig. 4). The method of randomisation to type of device will be by permuted blocks of varying size and stratified by key variables that might influence adherence with the device (potential confounders for the secondary outcome being evaluated for this comparison): site, age, and gender. The ratio will be adapted depending on adherence with submission of images that are of sufficient quality for teledermatology reporting. After 60 participants have been randomised into the intervention group, we will measure the proportion of intervention participants who have had an image reported on at one month after their baseline images (post randomisation) were due. If there is > 30% absolute difference in the proportion of intervention participants who have had an image reported on, participants who are subsequently enrolled will be randomised 2:1 to the dermatoscope model where more participants had an image reported on. If there is > 50% absolute difference in the proportion of intervention participants who have had an image reported on, then all participants who are subsequently enrolled will be randomised to dermatoscope model where more participants had an image that was reported on.

### Concealment mechanism {16b}

Participants will be randomised using the University of Sydney’s NHMRC Clinical Trials Centre’s Interactive Voice Response System (IVRS) which is a centralised telephone randomisation service. Allocation concealment will be ensured as the service will not provide the randomisation code until the patient has been recruited into the trial.

### Implementation {16c}

After participants have successfully completed the active run-in phase, the site coordinator will telephone the IVRS to perform random allocation and record the assigned treatment arm. The site coordinator will then distribute control and intervention packages to the participants.

### Assignment of interventions: blinding

#### Who will be blinded? {17a}

Blinding of participants, site staff and treating clinicians will not be possible in this trial. However, the teledermatologist who assesses the images submitted by participants in the intervention group will be blinded to the patient’s identity. For the primary outcome, the trial dermatopathologist will review the histopathology of all new melanoma diagnoses made during follow up blinded to study group allocation, and the endpoint adjudication committee will review the classification of clinic visit as routinely scheduled or as fast-tracked unscheduled blinded to study group allocation. Where possible, the endpoint adjudication committee will also review other secondary outcomes, blinded to study group allocation.

#### Procedure for unblinding if needed {17b}

The design is open label with only outcome assessors being blinded so unblinding will not occur.

### Data collection and management

#### Plans for assessment and collection of outcomes {18a}

##### Demographic and clinical information

At baseline, demographic and other risk factor information including age, sex, Indigenous status, non-English language background, marital status, children, occupation, income, highest education level, postcode, brand of smartphone owned, baseline use of digital technology/internet, risk of a subsequent new primary melanoma and keratinocyte cancer [48–50] and personal history of diagnosed depression or anxiety will be collected using standardised items from the Australian Census questionnaire and other instruments, where appropriate. Clinical information including time since first melanoma diagnosis, and characteristics of prior melanoma(s) including AJCC sub-stage and site, will be retrieved from administrative datasets at the clinics.

##### Questionnaires

During the active run-in phase participants will complete a baseline questionnaire and follow up questionnaires will be completed at 6 months and 12 months after randomisation. These questionnaires will collect data on SSE practice and beliefs [42], level of fear of new or recurrent melanoma [10], general stress, anxiety and depression (DASS-21) [43] and acceptability of reducing scheduled clinic visit frequency. Participants will complete questionnaires online through REDCap. Access codes and reminders will be sent to participants via email and SMS.

##### Patient diaries

Patient out of pocket and health system costs associated with each arm of the trial will be estimated using an online monthly resource use diary [51, 52]. The diary will be used to document and measure health service use - such as melanoma clinic visits, other health practitioner consultations, imaging and other tests, hospital visits, and time taken to take and submit images for teledermatology. We will ask patients to report the number of trips made to their melanoma follow-up clinic and the mode of transport used. We will also ask patients to report any visits to other physicians for a skin lesion excision or other skin cancer-related procedures. The time each patient spends to take and submit images, and check teledermatology reports (intervention group) will be recorded. The diary will also document informal caregiver (carer) days out-of-role (including paid and unpaid work), travel costs and direct carer costs.

##### Images and teledermatology reports

At baseline and 3 monthly intervals to 12 months post randomisation, intervention participants will be asked to take images using the mobile dermatoscope attached to their phone. They will use the corresponding app for the dermatoscope on their phone to submit the images to the corresponding web-based platform where the teledermatologist, site coordinator and research study staff can view them. Teledermatologists will use the same web platform to make and submit their reports on the lesions, which are then relayed to participants through the smartphone app. The teledermatology reports are also stored on the web platform for access by the research study staff.

##### Pathology

The research study staff will review participants’ medical records to collect and upload data including documents confirming eligibility (pathology reports and/or doctors’ letters) into REDCap. They will also confirm the number of skin lesions biopsied or removed and the number of melanomas and keratinocyte skin cancers diagnosed. The research study staff will upload source information including letters and pathology reports associated with excisions. After completion of the trial, any melanomas diagnosed during the trial will be reviewed by an expert dermatopathologist who is blinded to study arm, to confirm or refute the diagnosis. These reviews will be stored on REDCap.

##### Linkage

At completion, trial data will be linked with site databases for a schedule of clinic appointments, procedures, and pathology, and with the New South Wales (NSW) Cancer Registry and the Medicare Benefits Schedule claims database.

### Plans to promote participant retention and complete follow-up {18b}

#### Participant retention and withdrawals

We will promote participant retention through regular contact with the research study staff, with SMS text, email, or phone reminders to complete study activities. We will tabulate the number of patients whose consent for trial participation is withdrawn by the participant and those who are withdrawn by the research study staff due to loss to follow-up or if they move outside of NSW. Participants may choose to withdraw from active follow-up but consent to the ongoing passive collection of data (clinic, Cancer Registry and Medicare Benefits Scheme claims database) during the follow-up period. We will present descriptive summaries for the number of people who withdraw or are lost to follow-up, with present separate results for each category (type of withdrawal and loss to follow-up).

##### End of study

Active participation will continue for 12 months following randomisation or until all trial tasks have been completed (end dates will be staggered for individual participants and they will not be able to submit data after they have finished the 12 months follow up). We will collect linked data from the clinics, Cancer Registry and Medical Benefits Scheme for a further 12 months post-trial (i.e. 24 months post-randomisation).

### Data management {19}

Data management procedures are detailed in the data management plan included as Supplementary file 2.

### Confidentiality {27}

Research data will be stored in accordance with the University of Sydney’s Research Data Management Policy and Research Code of Conduct and will be stored on University managed and/or sanctioned storage infrastructure. Data will be secured via a personal login and data elements restricted by role at the direction of the Chief Investigator. After data collection, all identifiers such as participant names will be removed and replaced by a code. Electronic data will be re-identifiable for the duration of the project. Participant contact information (phone number and email address) will be stored in a quarantined area on REDCap, only visible to members of the research team who require it for study-related contact. This restriction will be built into REDCap user roles. Personal identifiers will be removed at trial completion, and only non-identifiable data will be stored subsequently.

### Statistical methods

#### Statistical methods for primary and secondary outcomes {20a}

A detailed Statistical Analysis Plan will be published separately with a full description of the statistical methods that will be used for analysing the primary and secondary outcomes, planned additional assessments including subgroup and sensitivity analyses, and methods to manage missing data (manuscript in preparation). All analyses will adhere to the intention-to-treat principle, unless otherwise stated. As a secondary analysis, we will also estimate the effect that would have been observed had all participants adhered to the protocol.

##### Primary outcome

We will use generalised linear models to investigate the difference between patient-led and clinician-led surveillance on the proportion of participants with a subsequent new primary or recurrent melanoma diagnosed through an unscheduled visit. We will present the proportion of participants with the primary outcome in each randomised group, and the between-group difference in proportions, along with the *p* value and 95% confidence interval (CI). Unadjusted and adjusted analyses will also be presented. For the latter, we will include baseline measurements of important risk factors for new or recurrent melanoma as covariates in the model. These will include age, sex, specialist/GP clinic, melanoma substage, subsequent new primary melanoma risk score [48] and dysplastic nevus syndrome. We will check the appropriate assumptions for the model. The adjusted and unadjusted analysis will be presented as an odds ratio along with the 95% CI and *p* value.

##### Secondary outcomes

We will assess the effect of patient-led and clinician-led surveillance on the secondary outcome of time to diagnosis of a skin cancer (melanoma or keratinocyte cancer), using Cox proportional hazards models. We will present unadjusted and adjusted analyses. For the latter, we will include the same covariates as for the primary outcome (important prognostic variables for outcome event). We will check the proportional hazards assumption using visual inspection of plots (including Schoenfeld residuals) and corresponding test statistics. Other assumptions to be checked include if there is non-informative censoring and if there is a secular trend. All participants will continue to be followed up unless they have withdrawn their consent or moved interstate. In the case that they have withdrawn their consent or moved interstate, participants will be censored at the last available follow-up. The unadjusted and adjusted hazard ratios with 95% CI and *p* values will be reported. If the assumptions including the proportional hazards assumptions are violated, these will be addressed as required. The time to diagnosis will also be analysed allowing for competing risk of death.

The appropriate generalised linear model will be used to assess the remaining secondary outcomes. In general, Poisson regression will be used for count variables, logistic regression for proportions and multiple linear regression for continuous outcome variables. We will assess effects on continuous outcomes (adherence with SSE; thoroughness, confidence, beliefs, attitude and knowledge of SSE; level of fear of new or recurrent melanoma; general anxiety, stress and depression; and acceptability of reducing the frequency of routinely scheduled clinic visits) by including baseline measurement of the outcome as well as other relevant prognostic variables as covariates in the multiple linear regression model. For example, for the outcome: level of fear of new or recurrent melanoma severity, we will include baseline measurement of FCRI severity subscale and personal history of depression or anxiety as covariates. We will estimate the between-group difference in change from baseline for each outcome, together with 95% CI and *p* values. We will check the appropriate model assumptions and if any are violated, then we will use other generalised linear models.

#### Costing analysis

Using a societal perspective, we will identify, measure and value all resources used to estimate the costs to patients, informal caregivers, to the health system, and to the environment (carbon emissions using relevant methods) for the intervention and control groups [35]. All costs associated with SSE, skin surveillance and management of newly identified lesions, out of pocket costs, opportunity costs and carbon costs will be included [11]. A detailed data health economics analysis plan will be developed and published separately.

##### Nested qualitative study

The nested qualitative study protocol is included in Supplementary File 3 and analysis is briefly outlined here. We will conduct telephone interviews at baseline, and at 6 and 12 months follow up among a sub-sample of patients and clinicians to allow qualitative evaluation of the intervention. We will invite participants who were randomised to both the intervention and control arms (controls at 12 months only) who indicated their interest in taking part in an interview to explore, in-depth, their experiences in the trial. A longitudinal format will be applied to interviews with intervention arm participants (see Supplementary File 3). Participants who withdraw from the intervention group will be approached for an interview also. Interviews will be audio-recorded and transcribed verbatim. Interviews with clinicians (*n* = 5–10) will explore their experience of the benefits and limitations of the intervention. The study will take a phenomenological perspective and will use Framework Analysis [53], a matrix-based method of thematic analysis which has been used successfully in other studies of early detection technologies [54, 55].

##### Dermatoscope technology comparison sub-study

We will compare performance of the two models of mobile dermatoscope (cross-polarised light dermatoscope or natural light dermatoscope) on the following outcomes:

1. Quality of image as reported by teledermatologists.
2. Adherence measured by the proportion of participants who have an image reported on at each of the 3 monthly submission points.
3. Participant satisfaction.
4. Protocol deviations, adverse events and device deficiencies related to use of the dermatoscope, app or teledermatology platform (for example, dermatoscope breakage, incorrect storage or transmission of images, delayed transmission of images).

We will measure these outcomes throughout the active trial period and analyse them using an appropriate statistical model that allows for repeated measures. The combination of adherence with the technology and quality of images will also be used for adaptive randomisation to protect against poor performance of one dermatoscope (see section 16).

### Interim analyses {21b}

Interim analyses will be conducted by an independent statistician after 33% of trial participants (approximately 200) have been recruited and, following this, after every 6 months. The interim analyses will be done masked to randomised groups and will estimate the frequency of the primary outcome to assess assumptions made for the sample size calculations, as well as the number of participants recruited, withdrawals and adverse events. Separate to the interim analyses described above, there will be an analysis as part of the adaptive randomisation after 60 participants have been randomised to the intervention arm (as described in Section 16). This will be limited to analysis of the device-related outcomes in the intervention arm only. There will be no comparison to the control group or estimation of effect on the primary outcome or other secondary outcomes for the adaptive randomisation. Any changes in the ratio of randomisation between the two types of device as a result of the adaptive randomisation will also be presented to the Data Safety Monitoring Committee (DSMC).

### Methods for additional analyses (e.g. subgroup analyses) {20b}

Subgroup analyses will be conducted to assess whether the effect of the intervention on the primary outcome (patient-led vs clinician-led surveillance) differs across the following patient characteristics (interaction between randomisation variable and patient characteristic):

- AJCC melanoma substage
- Risk of a subsequent new primary melanoma (1-year risk, continuous variable [48])
- Dysplastic naevus syndrome (yes or no)
- Age (continuous variable)
- Confidence in digital technology/digital health literacy

### Methods in analysis to handle protocol non-adherence and any statistical methods to handle missing data {20c}

Missing data will be accounted for by sensitivity analyses. Each variable will be assessed for missing data and if more than 10% of the data are observed to be missing on key variables, sensitivity analyses will be performed using an appropriate analysis method, such as multiple imputation, instrumental variable analysis or inverse probability weighting [56]. In addition, a per-protocol analysis will be conducted on the primary outcome to estimate the effect that would have been observed had all participants adhered to the protocol.

### Plans to give access to the full protocol, participant level-data and statistical code {31c}

Non-identifiable data and statistical code will be made available to other approved researchers to maximise the benefits that can be derived from the data. Access to NSW Cancer Registry (NSWCR) data requires the approval of the NSWCR Data Custodian. Access to the full protocol and to data (other than NSWCR data) may be requested via the CPI, Associate Professor Katy Bell.

### Oversight and monitoring

#### Composition of the coordinating centre and trial management committee {5d}

The Trial Management Committee (TMC) will coordinate the conduct of the trial and is independent of the trial’s financial sponsorship. TMC members include KB, MJ, AC, RT, LI, PG, RS, RPMS, VM, MD, JH, RM, Cl, DL, JT, JE and RAS. TMC responsibilities include protocol development; study planning, monitoring and progress; review of information from related research; and implementation of recommendations from other study committees and external bodies (e.g. Human Research Ethics Committee). The Expert Clinician Reference Group includes medical practitioners who have specific clinical roles in the trial (e.g. teledermatologists, treating doctors, pathologists), contributed to protocol development and provide ongoing clinical advice. The Trial Coordinating Team (TCT) consists of project coordinators, data managers and research assistants and is led by the Coordinating Principal Investigator (CPI). The TCT is responsible for the day-to-day management and governance of the trial.

#### Composition of the data monitoring committee, its role and reporting structure {21a}

The Data Safety Monitoring Committee (DSMC) will comprise three trial methodologists (senior statistician(s) and clinical trialist(s)) who are independent of the study team. The DSMC will meet to review each interim analysis and decide on any actions needed, which will be communicated to the Trial Management Committee.

#### Adverse event reporting and harms {22}

Please see Supplementary file 4.

#### Frequency and plans for auditing trial conduct {23}

##### Data monitoring and verification

Data quality assurance measures will include:

- Eligibility checks prior to randomisation
- Checks for unusual data patterns or trends
- Rates of recruitment, withdrawals, and losses to follow-up by site
- Checks for missing or invalid data on the electronic case report forms (eCRFs).
- Assessment of adverse event reporting rates compared between sites
- Image data review

Quality assurance processes are available on REDCap, and the data manager will verify data manually entered by the research study staff. Trial sites will regularly provide completed eCRFs to the data manager via REDCap. Copies of relevant documents for source verification and quality assurance will be requested, including imaging scans and histopathology reports. eCRF submission and query completion rates, as well as any issues related to protocol compliance, will be monitored.

##### On site and remote monitoring

Site monitoring will be scheduled annually for this study (subject to funding and recruitment rate and at the discretion of the TMC). Monitoring will address:

- Ongoing training
- Checks for understanding and adherence to trial protocol, Good Clinical Practice, and regulatory requirements
- Review of trial procedures (e.g. informed consent and safety reporting procedures, data capture, eCRF completion)
- Source data review to check quality and completeness
- Verification that resources and facilities remain adequate.

##### Auditing

This study is subject to audit by the TCT which could occur at any stage of the study. Sites will be informed in advance in writing, outlining the purpose and the scope of the audit should one occur.

### Plans for communicating important protocol amendments to relevant parties (e.g. trial participants, ethical committees) {25}

The protocol, statistical analysis plan, data safety management plan, informed consent forms, and participant education and recruitment materials have been reviewed and approved by Sydney Local Health District (RPAH Zone) Ethics committee New South Wales, Australia. Any subsequent modifications will be submitted for review, and annual safety and progress reports will be presented.

Potential protocol modifications will be submitted for approval by the above human research ethics committee before being implemented. All relevant parties will be notified including investigators, participants, and trial registries.

### Dissemination plans {31a}

The research team intends to disseminate outcomes broadly and at the earliest possible opportunity to allow access by other researchers and the wider community. Our findings will be made openly accessible in an institutional repository or other acceptable location (e.g. publisher website, subject repositories) within a 12-month period from the date of publication. Also, relevant stakeholders will be informed including participants, consumer groups, clinicians, the public and policymakers. The lay summary of findings will also be disseminated to consumer networks of Melanoma Institute Australia, the Australian Melanoma Consumer Alliance, associated charities, and Cancer Voices Australia. A summary of findings will be prepared for publication via social media platforms including Facebook and Twitter, newsletters, and press releases and through the University of Sydney’s School of Public Health website.

## Discussion

This randomised controlled trial will compare patient-led surveillance -with clinician-led surveillance for patients with a history of early-stage melanoma. It will generate evidence on whether patient-led surveillance can safely and effectively enable earlier detection of subsequent new primary or recurrent melanoma and keratinocyte cancers. It will also provide evidence of the acceptability of patient-led surveillance to patients and clinicians and report on the impact on health system resources and patient costs. The study is timely given the growing interest in digital healthcare services during the COVID-19 pandemic.

The investigators have considered the potential risks and impacts of COVID-19 on the conduct of this study. We plan to protect the health of participants and study personnel, while also minimising the impact of our study on the provision of health care, by implementing actions that reduce the requirement for face to face visits either temporarily or permanently. These actions will also protect the scientific integrity of the study.

Clinicians will limit patient face-to-face visits by using routinely scheduled visits to identify potential participants, provide an explanation of the research project to allow informed consent, and select a target lesion. These processes may also be achieved in a telehealth consultation where appropriate. We will minimise participant interactions with researchers. Recruitment packages will be emailed, and consent, questionnaires and diaries will be collected online through REDCap. Images will be uploaded into web-based platforms. Researchers will provide information and support by telephone and offer training on the intervention by video web conferencing. We will supply participants with dermatoscopes via couriers. Study staff will work remotely and meet via video web conferencing when public health advice recommend this. An issue related to the scientific integrity of the trial is that there may be more background telehealth use and alternative app use than previously. We plan to collect this information from participants in the baseline and follow-up questionnaires.

In conclusion, this study will contribute novel findings on evidence-based follow-up after treatment of early melanoma, to maximise patient wellbeing and the early detection of new or recurrent melanoma, while minimising costs to the patient and health system.

### Trial status

The protocol reported here is version 1.0 dated 19 February 2021. Trial recruitment will commence on approximately 1 July 2021 and will continue until the recruitment target is achieved and planned follow-up is completed (approximately 31 June 2023).

## Supplementary Information

**Additional file 1.**

**Additional file 2.**

**Additional file 3.**

**Additional file 4.**

## Data Availability

The materials used and datasets analysed during study will be available from the corresponding author on reasonable request.

## Abbreviations

AJCC: American Joint Committee of Cancer
app: Smartphone Application
ASICA: Achieving Self-Directed Integrated Cancer Aftercare
CPI: Coordinating Principal Investigator
eCRF: Electronic Case report form
DASS: Depression Anxiety and Stress Scale
DSMC: Data Safety Monitoring Committee
FCRI: Fear of Cancer Recurrence Inventory
GP: General Practitioner
HREC: Human Research Ethics Committee
MIA: Melanoma Institute Australia
PI: Principal Investigator
RCT: Randomised controlled trial
SSE: Skin self-examination
TCT: Trial Coordinating Team
TMC: Trial Management Committee

## References

[1] Australian Institute of Health and Welfare (AIHW). Cancer data in Australia. AIHW, Canberra. 2020. https://www.aihw.gov.au/reports/cancer/cancer-data-in-australia. Accessed 14 Jan 2021.

[2] Australian Institute of Health and Welfare. Cancer in Australia 2019. Cancer series no.119. Cat. no. CAN 123. AIHW, Canberra. 2019. https://www.aihw.gov.au/reports/cancer/cancer-in-australia-2019/summary. Accessed 14 Jan 2021.

[3] Australian Cancer Network Melanoma Guidelines Revision Working Party. Clinical practice guidelines for the Management of Melanoma in Australia and New Zealand. The Cancer Council Australia and Australian Cancer Network, Sydney and New Zealand Guidelines Group, Wellington. 2008. https://www.health.govt.nz/publication/clinical-practice-guidelines-management-melanoma-australia-and-new-zealand. Accessed 14 Jan 2021.

[4] Barbour A, Guminski A, Liu W, Menzies S, Morton R. What is the ideal setting, duration and frequency of follow-up for melanoma patients? In: Cancer Council Australia Melanoma Guidelines Working Party. Clinical practice guidelines for the diagnosis and management of melanoma. 2019. https://wiki.cancer.org.au/australia/Clinical_question:What_is_the_ideal_setting,_duration_and_frequency_of_follow-up_for_melanoma_patients%3F. Accessed 14 Jan 2021.

[5] Baade PD, Whiteman DC, Janda M, Cust AE, Neale RE, Smithers BM (2020). Long-term deaths from melanoma according to tumor thickness at diagnosis. Int J Cancer.

[6] Green AC, Baade P, Coory M, Aitken JF, Smithers M (2012). Population-based 20-year survival among people diagnosed with thin melanomas in Queensland. Australia J Clin Oncol.

[7] Lo SN, Scolyer RA, Thompson JF (2018). Long-term survival of patients with thin (T1) cutaneous melanomas: a Breslow thickness cut point of 0.8 mm separates higher-risk and lower-risk tumors. Ann Surg Oncol.

[8] Dummer R, Hauschild A, Lindenblatt N, Pentheroudakis G, Keilholz U, Committee EG (2015). Cutaneous melanoma: ESMO clinical practice guidelines for diagnosis, treatment and follow-up. Ann Oncol.

[9] Morton RL, Rychetnik L, McCaffery K, Thompson JF, Irwig L (2013). Patients' perspectives of long-term follow-up for localised cutaneous melanoma. Eur J Surg Oncol.

[10] Rychetnik L, McCaffery K, Morton R, Irwig L (2013). Psychosocial aspects of post-treatment follow-up for stage I/II melanoma: a systematic review of the literature. Psychooncology..

[11] Watts CG, Cust AE, Menzies SW, Coates E, Mann GJ, Morton RL (2015). Specialized surveillance for individuals at high risk for melanoma: a cost analysis of a high-risk clinic. JAMA Dermatol.

[12] Turner RM, Bell KJ, Morton RL, Hayen A, Francken AB, Howard K (2011). Optimizing the frequency of follow-up visits for patients treated for localized primary cutaneous melanoma. J Clin Oncol.

[13] Deckers EA, Hoekstra-Weebers JEHM, Damude S, Francken AB, ter Meulen S, Bastiaannet E (2020). The MELFO study: a multicenter, prospective, randomized clinical trial on the effects of a reduced stage-adjusted follow-up schedule on cutaneous melanoma IB–IIC patients—results after 3 years. Ann Surg Oncol.

[14] Moncrieff MD, Underwood B, Garioch JJ, Heaton M, Patel N, Bastiaannet E (2020). The MelFo study UK: effects of a reduced-frequency, stage-adjusted follow-up schedule for cutaneous melanoma 1B to 2C patients after 3-years. Ann Surg Oncol.

[15] Damude S, Hoekstra-Weebers JE, Francken AB, Ter Meulen S, Bastiaannet E, Hoekstra HJ (2016). The MELFO-study: prospective, randomized, clinical trial for the evaluation of a stage-adjusted reduced follow-up schedule in cutaneous melanoma patients-results after 1 year. Ann Surg Oncol.

[16] Belesova K, Heymann DL, Haines A (2020). Integrating climate action for health into covid-19 recovery plans. Bmj..

[17] Salas RN, Maibach E, Pencheon D, Watts N, Frumkin H (2020). A pathway to net zero emissions for healthcare. Bmj..

[18] Moynihan R, Johansson M, Maybee A, Lang E, Légaré F (2020). Covid-19: an opportunity to reduce unnecessary healthcare. Bmj..

[19] Sharma A, Jindal V, Singla P, Goldust M, Mhatre M (2020). Will teledermatology be the silver lining during and after COVID-19?. Dermatol Ther.

[20] Australian Bureau of Statistics. 2071.0 - Census of Population and Housing: Reflecting Australia - Stories from the Census, 2016. ABS, Canberra 2018. https://www.abs.gov.au/ausstats/abs@.nsf/Lookup/2071.0main+features1132016. Accessed 4 Feb 2021.

[21] Lim WY, Morton RL, Turner RM, Jenkins MC, Guitera P, Irwig L (2018). Patient preferences for follow-up after recent excision of a localized melanoma. JAMA Dermatol.

[22] Moore Dalal K, Zhou Q, Panageas KS, Brady MS, Jaques DP, Coit DG (2008). Methods of detection of first recurrence in patients with stage I/II primary cutaneous melanoma after sentinel lymph node biopsy. Ann Surg Oncol.

[23] Robinson JK, Reavy R, Mallett KA, Turrisi R (2020). Remote skin self-examination training of melanoma survivors and their skin check partners: a randomized trial and comparison with in-person training. Cancer Medicine.

[24] Morton RL, Francken AB, Dieng M, Balch CM, Atkins MB, Garbe C, Gershenwald JE, Halpern AC, Kirkwood JM (2020). Surveillance and follow-up of melanoma patients. Cutaneous Melanoma.

[25] Pollitt RA, Geller AC, Brooks DR, Johnson TM, Park ER, Swetter SM (2009). Efficacy of skin self-examination practices for early melanoma detection. Cancer epidemiology biomarkers &amp. Prevention..

[26] Coups EJ, Manne SL, Stapleton JL, Tatum KL, Goydos JS (2016). Skin self-examination behaviors among individuals diagnosed with melanoma. Melanoma Res.

[27] Yagerman S, Marghoob A (2013). Melanoma patient self-detection: a review of efficacy of the skin self-examination and patient-directed educational efforts. Expert Rev Anticancer Ther.

[28] Bell KJL, Mehta Y, Turner RM, Morton RL, Dieng M, Saw R (2017). Fear of new or recurrent melanoma after treatment for localised melanoma. Psychooncology..

[29] Rychetnik L, McCaffery K, Morton RL, Thompson JF, Menzies SW, Irwig L (2013). Follow-up of early stage melanoma: specialist clinician perspectives on the functions of follow-up and implications for extending follow-up intervals. J Surg Oncol.

[30] Murchie P, Allan JL, Brant W, Dennis M, Hall S, Masthoff J (2015). Total skin self-examination at home for people treated for cutaneous melanoma: development and pilot of a digital intervention. BMJ Open.

[31] Manahan MN, Soyer HP, Loescher LJ, Horsham C, Vagenas D, Whiteman DC (2015). A pilot trial of mobile, patient-performed teledermoscopy. Br J Dermatol.

[32] Koh U, Horsham C, Soyer HP, Loescher LJ, Gillespie N, Vagenas D (2019). Consumer acceptance and expectations of a mobile health application to photograph skin lesions for early detection of melanoma. Dermatology..

[33] A Research Study of Patient-led Surveillance Compared to Clinician-led Surveillance in People Treated for Localised Melanoma. ClinicalTrials.gov. 2020. https://ClinicalTrials.gov/show/NCT03581188. Accessed 7 Sept 2020.

[34] Bell KJ, Bossuyt P, Glasziou P, Irwig L (2015). Assessment of changes to screening programmes: why randomisation is important. Bmj..

[35] Malik A, Lenzen M, McAlister S, McGain F (2018). The carbon footprint of Australian health care. Lancet Planet Health.

[36] McAlister S, Barratt AL, Bell KJ, McGain F (2020). The carbon footprint of pathology testing. Med J Aust.

[37] Harris PA, Taylor R, Minor BL, Elliott V, Fernandez M, O'Neal L (2019). The REDCap consortium: building an international community of software platform partners. J Biomed Inform.

[38] Harris PA, Taylor R, Thielke R, Payne J, Gonzalez N, Conde JG (2009). Research electronic data capture (REDCap)—a metadata-driven methodology and workflow process for providing translational research informatics support. J Biomed Inform.

[39] Murchie P, Masthoff J, Walter FM, Rahman K, Allan JL, Burrows N (2019). Achieving Self-Directed Integrated Cancer Aftercare (ASICA) in melanoma: protocol for a randomised patient-focused pilot trial of delivering the ASICA intervention as a means to earlier detection of recurrent and second primary melanoma. Trials..

[40] Ngoo A, Finnane A, McMeniman E, Tan JM, Janda M, Soyer HP (2018). Efficacy of smartphone applications in high-risk pigmented lesions. Australas J Dermatol.

[41] Chuchu N, Takwoingi Y, Dinnes J, Matin RN, Bassett O, Moreau JF (2018). Smartphone applications for triaging adults with skin lesions that are suspicious for melanoma. Cochrane Database Syst Rev.

[42] Janda M, Youl P, Neale R, Aitken J, Whiteman D, Gordon L (2014). Clinical skin examination outcomes after a video-based behavioral intervention: analysis from a randomized clinical trial. JAMA Dermatol..

[43] Lovibond SH, Lovibond PF (1995). Manual for the depression anxiety stress scales.

[44] Janda M, Horsham C, Vagenas D, Loescher LJ, Gillespie N, Koh U (2020). Accuracy of mobile digital teledermoscopy for skin self-examinations in adults at high risk of skin cancer: an open-label, randomised controlled trial. Lancet Digital Health.

[45] Lim WY, Turner RM, Morton RL, Jenkins MC, Irwig L, Webster AC (2018). Use of shared care and routine tests in follow-up after treatment for localised cutaneous melanoma. BMC Health Serv Res.

[46] Dworkin SL (2012). Sample size policy for qualitative studies using in-depth interviews. Arch Sex Behav.

[47] Memari N, Hayen A, Bell KJ, Rychetnik L, Morton RL, McCaffery K (2015). How often do patients with localized melanoma attend follow-up at a specialist center?. Ann Surg Oncol.

[48] Cust AE, Badcock C, Smith J, Thomas NE, Haydu LE, Armstrong BK (2020). A risk prediction model for the development of subsequent primary melanoma in a population-based cohort. Br J Dermatol.

[49] Olsen CM, Pandeya N, Thompson BS, Dusingize JC, Webb PM, Green AC (2018). Risk stratification for melanoma: models derived and validated in a purpose-designed prospective cohort. J Natl Cancer Institute.

[50] Whiteman DC, Thompson BS, Thrift AP, Hughes MC, Muranushi C, Neale RE (2016). A model to predict the risk of keratinocyte carcinomas. J Invest Dermatol.

[51] Sanders GD, Neumann PJ, Basu A, Brock DW, Feeny D, Krahn M (2016). Recommendations for conduct, methodological practices, and reporting of cost-effectiveness analyses: second panel on cost-effectiveness in health and medicine. JAMA..

[52] Husereau D, Drummond M, Petrou S, Carswell C, Moher D, Greenberg D (2013). Consolidated health economic evaluation reporting standards (CHEERS) statement. BMJ..

[53] Ritchie J, Lewis J (2003). Qualitative research practice: a guide for social science students and researchers.

[54] Smith SK, Dixon A, Trevena L, Nutbeam D, McCaffery KJ (2009). Exploring patient involvement in healthcare decision making across different education and functional health literacy groups. Soc Sci Med.

[55] Waller J, McCaffery K, Kitchener H, Nazroo J, Wardle J (2007). Women’s experiences of repeated HPV testing in the context of cervical cancer screening: a qualitative study. Psychooncology..

[56] Hernán M, Robins J (2020). Causal inference: what if.

