# Supplementary material for: Can patient-led surveillance detect subsequent new primary or recurrent melanomas and reduce the need for routinely scheduled follow-up? A protocol for the MEL-SELF randomised controlled trial
Source: Trials. 2021 May 4;22:324. doi: 10.1186/s13063-021-05231-7 (PMC8096155; doi:10.1186/s13063-021-05231-7)
Supplement: Supplementary file 1 — Additional file 1. [file 13063_2021_5231_MOESM1_ESM.docx]

Supplementary file 1.

| **When is your next scheduled clinic visit?**  Options:  Enter date (dd/mm/yyyy)  No visit scheduled |
| --- |
| **Please indicate if you have noticed any of the following with this lesion (select all that apply):** |
| This is a new lesion |
| Lesion has not changed |
| Lesion is in a place that is hard for me to see on my own |
| This is a new lump or lymph node |
| Change in lesion size/shape  → If selected: ‘For how long has the lesion been changing?’ |
| Change in lesion colour  → If selected: ‘For how long has the colour been changing?’ |
| Lesion is bleeding  → If selected: ‘For how long has the lesion been bleeding?’ |
| Lesion is painful  → If selected: ‘For how long has the lesion been painful?’ |
| Lesion is itchy  → If selected: ‘For how long has the lesion been itchy?’ |
| Recent irritation/trauma to lesion  → If selected: ‘For how long has the lesion been irritated?’ |
| Other notes: (free text) |

Table 1. Patient history information

| **Recommendation**  **(only selected one appears at patient end)** |
| --- |
| Unfortunately the image quality is not sufficient for teledermatology review. Please retake the photo. |
| This lesion does not seem to be suspicious at this time. Please self-monitor and review with your doctor at your next scheduled visit |
| There is no immediate concern today, but this lesion should continue to be monitored through teledermatology. Please take another picture in _ months and submit for review. |
| We recommend that you book an appointment with your doctor to examine this lesion. Please call your melanoma clinic to make an appointment at the earliest opportunity [if possible add name of clinic and the phone number] |
| Other: (free text notes) |

Table 2. Teledermatologist recommendations
