# Supplementary material for: Can patient-led surveillance detect subsequent new primary or recurrent melanomas and reduce the need for routinely scheduled follow-up? A protocol for the MEL-SELF randomised controlled trial
Source: Trials. 2021 May 4;22:324. doi: 10.1186/s13063-021-05231-7 (PMC8096155; doi:10.1186/s13063-021-05231-7)
Supplement: Supplementary file 2 — Additional file 2. [file 13063_2021_5231_MOESM2_ESM.docx]

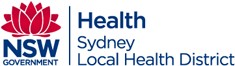


**Research Data Management Plan (RDMP template)**

The Data Management Plan can be used in a number of ways to assist with data management planning.

These include:

- To identify a series of issues and underlying questions that should be considered when planning a research project and initiate discussions within a project (team)
- For the development of a data management plan
- To raise awareness of good practice when planning for data management during the life cycle of a research project.

The RDMP is informed by the requirements set out in the Australian Code for the Responsible Conduct of Research, the UK Digital Curation Centre Checklist and current SLHD governance practices.

Use the template effectively to develop a data management plan by providing detailed and descriptive responses to the following questions. Please provide as much descriptive information as possible and indicate ‘Not Applicable’ (N/A) for those elements you determine are not relevant to your project.

| **Document History** | |
| --- | --- |
| Author of this document | Katy Bell |
| Author’s relationship to the  project/data | Chief Investigator |
| Date document created | 1/07/2020 |
| Document Version | Version 1 |
| Date document modified/updated | 16/02/2021 – minor updates to ensure consistency with protocol. |
| Date to review this document | In 12 months |
| Position or person  responsible for reviewing the plan, if applicable |  |

| **1.0** | **ABOUT THE RESEARCH PROJECT** | | |
| --- | --- | --- | --- |
| **1.0** | | Project title | MEL-SELF: A randomised controlled trial of patient-led surveillance compared to clinician-led surveillance in people treated for early-stage melanoma (stage 0/I/II) |
| **1.1** | | Project description | This RCT of patients who have had surgery for early-stage melanoma and are being followed up through regular scheduled clinics will compare patient-led surveillance (usual care + mobile dermatoscope + smartphone app + teledermatology + reminders + an educational booklet + scheduled and unscheduled clinic visits) with clinician-led surveillance (usual care + educational booklet + scheduled and unscheduled clinic visits) on detection rates for recurrent or new primary melanomas. We will also document how well participants are able to perform self-examination, their levels of melanoma-related anxiety, the number of skin lesions biopsied or removed, the number of non-melanoma skin cancers diagnosed, and the costs to the participant and the healthcare system. |
| **1.2** | | Date project commencing | 1 Jun 2021 |
| **1.3** | | Estimated date of project completion | July 2025 |
| **1.4** | | Name of Chief Investigator | Associate Professor Katy Bell |
| **1.5** | | Name/s of Partner Investigators | See Protocol |
| **1.6** | | Name of Supervisor (if applicable) | Not Applicable |
| **1.7** | | Primary contact for the data | Associate Professor Katy Bell |
| **1.8** | | Name of Department/Centre | The University of Sydney School of Public Health |
| **1.9** | | Lead partner organisation | Not Applicable |
| **1.10** | | Other partner organisations | Not Applicable |

| **2.0** | **FUNDING BODY** |
| --- | --- |
| Provide details of relevant funding bodies and their Grant application numbers. | |
| National Health and Medical Research Council (NHMRC) Project funding  Grant identification number APP1163054 | |

| **3.0** | **DATA TO BE PRODUCED** | |
| --- | --- | --- |
| Describe the type of data; its characteristics and features; the methods or processes for producing the data; expected file formats; use of existing or third-party data and any requirements associated with its use. | | |
| Type of data will be produced, collected, generated, or captured during the project? | | Personal data; Medical records; Publicly held database (Commonwealth); Publicly held database (State or local) |
| How will the data be captured, collected, or created? (describe process) | | Data that will be collected and used includes:   1. Consent forms will be stored electronically in Research Electronic Data Capture (REDCap) database which is hosted on the University of Sydney’s secure server. If required, hardcopy consent forms can also be uploaded into REDCap by the Site Coordinator. 2. Questionnaires   Participants will complete questionnaires online via REDCap. Access codes and initial reminders are sent via email or SMS through REDCap. Hardcopy questionnaires can also be completed as a back-up option (if participants fail to complete online after 2 reminders) and entered manually into REDCap by the Site Coordinator.   1. Diaries   Participants will complete diaries online into REDCap Hardcopy diaries may be completed as a back-up option and entered manually into REDCap by the Site Coordinator.   1. Site databases.   Clinic appointments, procedures and other information will be entered into REDCap by the Site Coordinator.   1. Pathology reports and letters   The Site Coordinators will upload de-identified source documents (histopathology reports and/or doctors’ letters) into REDCap. After completion of the trial, any melanomas diagnosed during the trial will be reviewed by an expert dermatopathologist, Prof Richard Scolyer. Site coordinators will upload de-identified versions of his reviews to REDCap.   1. Linked Data.   At completion, trial data will be linked with the following databases:   - 1. Site databases   2. NSW Cancer Registry   3. Medicare  1. Images   Intervention participants take images using the mobile dermatoscope on their phone. They use the MoleScope App on their phone to submit the images to the corresponding web-based platform (Dermengine) where the teledermatologist, site coordinator and designated project coordinator can view them. Teledermatologists use the same web platform to make and submit their reports which are relayed to participants through the smartphone App. The teledermatology reports are also stored on the web platform for access by site coordinator and designated project coordinator. These are secure web-based platforms which are based in Australia and meet privacy requirements under Australian law. Our CTSA legal agreement stipulates that an Australian IP address is used for the website. The site coordinator uploads de-identified reports into the relevant appointment form on REDCap as well.   1. Interviews (sub-study)   Interviews with participants and clinicians are recorded using an audio recorder. Transcripts and recordings will be uploaded into REDCap |
| What tools, instruments, equipment, hardware, or software will you use to capture, produce, collect or create the data? | | Electronic data will be collected and managed using REDCap  Images and Teledermatology reports will be stored on MoleScope (DermEngine) web-based platforms.  Interviews are recorded using an audio recorder and stored as an MP3 File (.MP3) in REDCap. |
| What are the expected file formats of the data that will be captured, produced, or created? | | Data downloads for REDCap will be as csv and SAS formats. Interviews are recorded and stored as an MP3 File (.MP3). |
| Are these file formats based on open standards, non-proprietary or widely used, documented, and supported? | | Yes, these are widely used. |
| Will the project use existing or third-party data as part of the investigation? | | Data will be obtained from Participant medical records, the NSW Cancer Registry and Medicare.  Linkage will be performed by the Centre for Health Record Linkage (CHeReL). |
| Are there any requirements for use of third-party data such as licensing conditions? | | Participant consent will be prospectively obtained for linkage with these databases, and approval from the data custodians will be sought. We have correspondence from Cancer Institute NSW showing support in principle for the cancer registry linked data analyses. |

| **4.0** | DATA DOCUMENTATION AND METADATA | |
| --- | --- | --- |
| Provide details of any supporting information to be developed or documented; any metadata standard, controlled vocabularies or ontologies that will be used to describe the data; quality assurance processes (calibration, validation, etc.) to be applied to the data; and any processes that will be followed for documenting or organizing the data such as file name conventions, directory structures, etc. | | |
| What supporting information/documentation will you create to enhance understanding of the data? e.g., codebooks, data dictionaries, data definitions, publications, websites. (please attach data dictionary as appendix to this document) | | REDCap database contains a Data dictionary and code book. These will be adapted for study staff to provide clear definitions of data elements to enhance understanding of data.  The data dictionary will be made available once the database construction is finalised. |
| Are you using any metadata standards, controlled vocabularies, or ontologies to describe the data? | | No – No data sharing envisaged |
| Are there any Quality Assurance processes that could be applied to your data? e.g., calibration, validation, transcription, peer- review etc. | | Yes. Quality assurance processes are available on REDCap. The data manager will verify data that has been manually entered by the site coordinator.  Site monitoring is scheduled annually for this study |
| What processes will be established and followed to document and organise data? i.e. version control, filename conventions, directory structures etc. | | Version Control and filename conventions will be employed to document and organise the data. |

| **5.0** | DATA STORAGE AND SECURITY | |
| --- | --- | --- |
| Describe data storage and security arrangements: estimated size/amount of data; the location of where the data will be stored; the location of where the data will be backed-up to; frequency of back- up procedures and person responsible; how access to the data will be managed; any security or restriction issues relating to access or storage; and details of any physical or non-digital outputs that need to be stored including their location. | | |
| How much data are you likely to collect/generate throughout the project? (numbers of records/patients/surgeries or sizes of files /sizes of databases)  Where will the data be stored during the project? | | 600 participant records will be generated through the project and stored on REDCap.  The maximum number of images that could be collected would be 12000, if all patients submit 8 images at 0, 3, 6, 9 and 12 months.  Approximately 40 interview recordings and transcripts will be stored in REDCap. |
| Will your data be backed up, by whom, how often, where? | | Data is automatically backed up on REDCap on The University of Sydney secure servers.  Images are backed up by the companies that provide the teledermatology web-platforms. Site coordinators will periodically download data from the teledermatology platforms (e.g., dashboard data and teledermatology reports) and upload these into REDCap. |
| How will access to the data be managed during the project? | | Participant contact information (phone number and email address) will be stored in a quarantined area on REDCap, only be visible to members of the research team who require it in order to contact participants to complete study assessments and study related required contact. This restriction will be built into to REDCap user roles. Research data will be stored in accordance with the University of Sydney’s Research Data Management Policy and Research Code of Conduct and will be stored on University managed and/or sanctioned storage infrastructure. Data will be secured via a personal login and data elements restricted by role at the direction of the Chief Investigator. |
| Are there any commercialisation, ethical or confidentiality restrictions relating to accessing or storing the data during the project? | | Yes, there are ethical and confidentiality issues related to the management of personal and health information.  We have minimised the risk to personal privacy by ensuring:   - Only researchers involved in data analysis will have access to site record health data. - Data will be stored securely on University managed and/or sanctioned storage infrastructure. Data will be secured via a personal login. - Researchers analysing the data will not be in possession of any personally identifying information. |
| Is there any non-digital data or outputs that the project will generate? Where will these outputs be stored? | | Hard copy materials will be uploaded into REDCap. Hard copy source materials will be filed securely at study sites in line with their data governance procedures. |

| **6.0** | ETHICS, COPYRIGHT, IP AND AUTHORSHIP | |
| --- | --- | --- |
| Provide information on Ethics, copyright and IP arrangements : methods used to manage sensitive, confidential or private information; details of any restrictions due to ethical or privacy considerations on the data; information for consent forms relating to retention of the data and protection of privacy and confidentiality and steps taken to manage these (de-identification, etc.); details of any agreements reached with partner organizations concerning ownership of the data; any copyright or licensing restrictions; or legislative regulations or requirements associated with collecting data from/sending to countries/locations outside of Australia. | | |
| Does/will the data contain sensitive, confidential or personal information? If yes, what methods will be used to protect the data e.g. encryption, password restrictions etc. | | Yes, it will contain participant personal and medical information.  After data collection, all identifiers such as participant names and date of birth will be removed and replaced by a code. Electronic data will be re-identifiable for the duration of the project. Personal identifiers will be removed at completion and only non-identifiable data will be stored.  Data will be password-protected on a secure REDCap project, requiring a valid REDCap account that is authorised to access the project. |
| Are there ethical/privacy considerations surrounding the ability to share/publish the research data outside the immediate research team? | | Only non-identifiable research data will be published. We will publish aggregate data and will ensure that any subgroup analyses do not allow identification of individuals. |
| If intending to share any part of the data, do your participant consent forms include information about intentions for sharing, retention of data and steps taken to protect participants privacy and confidentiality? | | Yes, refer to Participant Information Sheet for details. |
| What steps will be taken to protect privacy and confidentiality? e.g., de-identification, re-identification or anonymising data. | | After data collection, all identifiers such as participant names will be removed and replaced by a code. Electronic data will be re-identifiable for the duration of project. Personal identifiers will be removed at completion and only non-identifiable data will be stored.  Data will be password-protected on a secure REDCap project, requiring a valid REDCap account that is authorised to access the project. |
| Has an agreement about the ownership of research data and primary materials been reached between partner institutions? Provide details. If the agreement is in writing, add as appendix to this RDMP | | The University of Sydney owns all data generated from the study. We have a draft *Agreements for Clinical Trial Services* with MetaOptima. |
| Are there likely to be any copyright restrictions that will apply to the data? | | No |
| Will the data be collected in or transported to another country or area outside of Australia? Are there any legislative requirements to meet? | | No |
| Have you considered and discussed authorship? Who are the intended authors of any products of the research project? | | Please refer to the protocol for the full list of potential authors.  Further potential authors may be considered. For any publication, all authors must meet the criteria set out by the International Committee of Medical Journal Editors (ICMJE). |

| **7.0** | ACCESS, SHARING, REUSE OF DATA | |
| --- | --- | --- |
| Provide information on access, sharing and reuse arrangements including : what data or non digital outputs will be retained on completion of the project; where will these be stored; will some/all of the data be shared or published; any restrictions that negate sharing or re-use of the data; any requirements for mediating access to the data; what supporting information will be available to assist with interpretation of the data; what processes or steps will be taken to protect privacy and confidentiality; intent to deposit in data repository or archive; how soon after completion of the project can the data be shared; and any costs associated with making the data available for sharing or re-use. | | |
| Will part/all of the data be retained on completion of the project? Where will this data be stored? | | Research data and related materials will be managed by the University of Sydney and retained in accordance with the University’s Research Data Management Policy and Research Code of Conduct and stored on University managed and/or sanctioned storage infrastructure. |
| Where will non-digital data be stored post project? | | Any hard copy materials collected by the site coordinators will be archived at the site. Any hard copy materials collected by the research team will be protected during and after the project as they will be stored in locked filing cabinets in locked offices in the Edward Ford Building (A27) at the University of Sydney. |
| How will access to the data be managed post project? | | Access to the data will be arranged by contacting the Chief Investigator, Associate Professor Katy Bell. |
| Do you plan to share some/part of the data post project? Will the data be deposited with an archive or repository or published on the web? | | Publications will be disseminated broadly and at the earliest possible opportunity to allow access by other researchers and the wider community. Our findings will be made openly accessible in an institutional repository or other acceptable location (e.g., publisher website, subject repositories) within a 12-month period from the date of publication.  Non-identifiable data will be made available to other researchers to maximise the benefits that can be derived from the data.  Electronic data will be archived in a non­identifying format to allow data sharing with approved researchers, in accordance with the data sharing requirements of the major medical journals and granting bodies. |
| Are there any restrictions placed on sharing/reuse of some/all of the data? | | Only de-identified data will be shared. |
| Can these be managed by setting mediated access to the data? e.g., access to the data must be negotiated via Chief Investigator. | | Access to the data must be negotiated via the Chief Investigator, Associate Professor Katy Bell. |
| What supporting information to assist with interpretation of the data will be made available? How will the information be made available? | | A data dictionary and code book will be available. It will be made available in Excel format either .csv or .xlsx format. |
| How will you ensure that identified processes or steps taken to protect privacy and confidentiality will be achieved prior to completion of the project and sharing of the relevant data. | | The University of Sydney will have ownership of data which will be converted into a non-identifiable form after the completion of the trial.  The consent form specifies that data may be used for research that is closely related to this research project. |
| When will the data be shared post project? e.g., immediately, 3 months, 6 months,1 year. | | Within 12 Months of final publication of all study results |
| Is there likely to be any costs associated with making the data available for sharing or re-use? | | Nil |

| **8.0** | DATA RETENTION AND DISPOSAL | |
| --- | --- | --- |
| Provide information on data retention and disposal, including how long the data should be retained (in line with University Policy, State Records Act 1998, and/or Funding Body requirements; the disposal date and data disposal approval process that will be followed, in line with University Policy. | | |
| Is there likely to be any costs associated with making the data available for sharing or re-use? | | Nil |
| How long the data should be retained for?  i.e., permanently, 5 years, 7 years, 20 years, etc. | | Electronic data, in a non-identifiable format, and hard copies will be stored for a minimum of 15 years. |
| If disposing of data, outline how will you handle the disposal of sensitive, confidential data | | After the required storage period, electronic data will be destroyed by removing and deleting all files from the University secure server. Hard copies will be destroyed using University shredding facilities. |

| **9.0** | PRESERVATION & ARCHIVING | |
| --- | --- | --- |
| Provide information describing preservation and archiving arrangements, including: the sustainable file formats that will be used for long term access; descriptive information details the organization and structure of the data and supporting information that will be made available with the data for re-use and interpretation; the person or position responsible for managing long-term access to the data; and any expected costs associated with long term storage of the data. | | |
| Will the final format of the data files be in a sustainable format supporting long term access? i.e., based on open-source standards, non-proprietary. | | Yes, csv format. |
| Will the supporting documentation be stored with the data to enable future interpretation? | | Yes |
| Who is responsible for maintaining the data after the research project is complete? e.g., Chief Investigator, data manager, research assistant. | | Chief Investigator |
| Are there likely to be any costs associated with the long-term storage of the data? | | Nil |
