# Supplementary material for: Can patient-led surveillance detect subsequent new primary or recurrent melanomas and reduce the need for routinely scheduled follow-up? A protocol for the MEL-SELF randomised controlled trial
Source: Trials. 2021 May 4;22:324. doi: 10.1186/s13063-021-05231-7 (PMC8096155; doi:10.1186/s13063-021-05231-7)
Supplement: Supplementary file 3 — Additional file 3. [file 13063_2021_5231_MOESM3_ESM.docx]

**Acceptability of and satisfaction with patient-led melanoma surveillance using a teledermatology smart phone application – a qualitative evaluation of patient and clinician attitudes and practices.**

**Objectives**

To understand patients’ and clinicians’ acceptability of and satisfaction with the patient-led melanoma surveillance intervention which includes use of a teledermatology smart phone app and dermatoscope; the ways in which patients used the intervention, how clinicians experienced the intervention, and reasons for such practices and interactions. The qualitative evaluation will inform scale up of the intervention into routine clinical practice.

**Study design and setting**

A longitudinal study design will be used to capture changes in attitudes and behaviour of intervention arm participants over time. At baseline, 6 months and 12 months after randomisation, semi-structured interviews will be conducted with (a) patients in the intervention arm. If and when a participant is lost to follow up or declines to be interviewed again, another participant will be invited for interview in their place. Interviews with (b) clinicians who were involved in recruitment, treatment or interpretation of images collected with the dermatoscope by patients in the intervention arm, will be conducted at baseline, 6 months and 12 months. Interviewed clinicians may differ at each time point. Semi-structured interviews at 12 months after randomisation will also be conducted with (c) control arm patients. Interviews with control arm participants will enable future realist evaluation. Additionally, we aim to interview (d) patients who withdraw from the study at any point after being randomised to the intervention group. We aim to conduct these interviews as close as possible to the time of dropout.

**Aims**

- Assess patient acceptability of and satisfaction with smart phone app supported skin self-examination and teledermatology.
- Assess how patients used the smart phone app and dermatoscope.
- Explore benefits and limitations of the digitally supported patient-led surveillance intervention as experienced by clinicians.
- Determine which components of the intervention and its implementation into clinical care need to be adjusted or changed for scale up into routine clinical practice.

**Study population**

Adult participants who have been treated for a first primary melanoma, stage 0/I/II, and who are undergoing regular melanoma follow-up at recruiting melanoma and skin cancer clinics in NSW.

Clinicians involved in the care of the trial participants described above.

**Eligibility**

Inclusion and exclusion criteria for patients are the same as those for the main trial.

Inclusion criteria (clinician):

Are a treating clinician of an intervention arm study participant or was involved in their recruitment or assessment of images taken with the smart phone application.

**Sample selection**

A sub-set of participants recruited to both study arms and all clinicians of intervention arm patients will be approached and invited to take part in the nested qualitative study. Up to 45 semi-structured interviews will be held with patients, and up to 10 semi-structured interviews will be held with clinicians. A sample size of 30 participants in the qualitative study is a well-accepted sample size commonly sufficient to reach saturation in themes and topics.(1) We have increased the sample size to a maximum of 45 to accommodate for interviews with intervention arm participants at three time points, control arm participants and intervention group dropouts. The sample will be selected using a purposive approach in order to capture indicators of diversity that characterise study participants, such as residing in urban and rural areas, age, sex, and those who are adherent and non-adherent with SSE. If required, ongoing selection of participants for interview will be driven by emerging theoretical concerns.

**Recruitment**

Study staff will invite patients and clinicians via email initially, and phone thereafter if a response is not received within two weeks. The MEL-SELF main trial PIS explains that intervention and control arm study participants may be involved in a qualitative interview if they wish. Patient and clinician contact details will be taken from information held by the main MEL-SELF clinical trial.

**Data collection**

Interviews will be conducted via telephone or teleconferencing. Semi-structured interviews will be conducted by trained research staff. The interviews will be informed by flexible topic guides developed by the multidisciplinary study team, covering key areas of investigation. As is appropriate for qualitative research, analysis will be conducted iteratively and will inform the focus of ongoing data collection.

To maximise the engagement and confidence of the participant in the qualitative research process, interviews will be arranged to be conducted at a time and that is convenient to the participant, we anticipate this will be primarily over the phone. Interviews will last approximately 30-45 minutes. Permission to record interviews will be a part of the consent process. Participants will be assured of the confidentiality of their interview accounts. Pseudonyms will be used throughout subsequent reporting of the interviews.

**Data analysis**

All qualitative interviews will be audio recorded and transcribed verbatim by a professional transcription service. Transcripts will be checked by the research team for accuracy and clarity. Interview summaries will be written by the interviewer and shared with study investigators, as soon as is feasible after each interview. This will form the basis for the team’s discussions to perform the initial analyses, refine the topic guide and inform ongoing sample selection.

Data analyses will be conducted by the research staff in collaboration with the investigator team. The study will take a Phenomenological perspective and will use Framework Analysis,(2) a matrix based method of thematic analysis which has been used successfully in numerous screening studies.(3, 4) This will be accompanied by the iterative development of analytical memos which will inform comprehensive analysis of the data by case to examine the attitudes towards, and experiences and practices using the teledermatology app and the dermatoscope. Analysis will continue until thematic saturation has been reached. The focus of analysis will be on providing insights to understand what determines more effective and less effective use of the teledermatology app, the dermatoscope, and teledermatology in general, for patients and clinicians, to ultimately inform integration of the intervention into routine clinical practice.

**Ethical considerations**

Written consent will be sought from interview participants, including consent to audio recording. Qualitative data will be stored in accordance with the University of Sydney’s Research Data Management Policy and Research Code of Conduct and will be stored on University managed and/or sanctioned storage infrastructure. After data collection, all identifiers such as participant names will be removed and replaced by a code. Electronic data will be re-identifiable for the duration of the project. Re-identifiable information will be stored in a password protected area and only accessible by members of the research team.

1. Dworkin SL. Sample size policy for qualitative studies using in-depth interviews. Arch Sex Behav. 2012;41(6):1319-20.

2. Ritchie J LJ. Qualitative Research Practice: A Guide for Social Science Students and Researchers. Sage, editor. London: Sage; 2003.

3. Smith SK, Dixon A, Trevena L, Nutbeam D, McCaffery KJ. Exploring patient involvement in healthcare decision making across different education and functional health literacy groups. Soc Sci Med. 2009;69(12):1805-12.

4. Waller J, McCaffery K, Kitchener H, Nazroo J, Wardle J. Women's experiences of repeated HPV testing in the context of cervical cancer screening: a qualitative study. Psychooncology. 2007;16(3):196-204.
