# Supplementary material for: Can patient-led surveillance detect subsequent new primary or recurrent melanomas and reduce the need for routinely scheduled follow-up? A protocol for the MEL-SELF randomised controlled trial
Source: Trials. 2021 May 4;22:324. doi: 10.1186/s13063-021-05231-7 (PMC8096155; doi:10.1186/s13063-021-05231-7)
Supplement: Supplementary file 4 — Additional file 4. [file 13063_2021_5231_MOESM4_ESM.docx]

**Adverse event reporting and harms {22}**

## Definitions

The trial will use the following definitions as recommended by the National Health and Medical Research Council Safety monitoring guidelines for clinical trials involving therapeutic devices.

**Adverse Event (AE)**

Any untoward medical occurrence, unintended disease or injury, or untoward clinical signs in participants, users, or other persons, whether or not related to the investigational medical device.

**Adverse Device Effect (ADE)**

Adverse event related to the use of an investigational medical device. In this trial an example of an ADE may be increased anxiety associated with the intervention.

**Device Deficiencies**

Inadequacy of a medical device with respect to its identity, quality, durability, reliability, safety or performance.

Device deficiencies related to teledermoscopy and use of the smartphone app and dermatoscope may include:

- Issues related to timeliness of assessment such as problems with transmission of the image to the dermatologist and delayed result communication.
- Device faults and repairs.
- Issues related to difficulty of use

**Serious Adverse Event (SAE)**

An adverse event that:

- Led to death
- Led to serious deterioration in the health of the participant, that either resulted in:
  - a life-threatening illness or injury, or
  - a permanent impairment of a body structure or a body function, or
  - in-patient or prolonged hospitalisation, or
  - medical or surgical intervention to prevent life-threatening illness or injury or permanent impairment to a body structure or function.

**Serious Adverse Device Effect (SADE)**

An adverse device effect that has resulted in any of the consequences characteristic of a serious adverse event.

**Significant Safety Issue (SSI)**

A safety issue that could adversely affect the safety of participants or materially impact on the continued ethical acceptability or conduct of the trial.

**Unanticipated Serious Adverse Device Effect (USADE)**

Serious adverse device effect which by its nature, incidence, severity, or outcome has not been identified in the current version of the risk analysis report.

**Anticipated serious adverse device effect (ASADE)** is an effect which by its nature, incidence, severity, or outcome has been identified in the risk analysis report.

**Urgent Safety Measure (USM)**

A measure required to be taken in order to eliminate an immediate hazard to a participant’s health or safety. Note: Action to address this type of significant safety issue can be instigated by either the investigator or sponsor and can be implemented before seeking approval from HRECs or institutions.

## Collection

Data related to harms and device deficiencies will be collected through multiple sources by the site coordinators, including contacts they have with the patient as part of follow-up related to intervention activities, clinic visit records and doctors’ letters.

## Assessment

The site PI should assess all adverse events and act on these as clinical care dictates. Each adverse event will be evaluated for:

- Seriousness: An assessment of whether the adverse event meets the definition of a SAE.
- Causality: A clinical assessment of whether there is a reasonable causal

relationship between the adverse event and the use of the investigational medical device.

- Expectedness: An assessment of whether the adverse reaction or adverse

device effect is consistent with information previously known.

## Reporting

All AEs, ADEs and device deficiencies occurring from enrolment into the study until completion of trial activities by the participant will be recorded. These will be reviewed by the TMC, the DMSC and included in the annual report to the Ethics committee.

Principal investigators should ensure that serious adverse events are reported to the TCT and CPI within 24 hours. SAEs will be reported according to NHMRC and NSW Health guidelines and are outlined in tables and flowcharts in the appendix. Adverse events (AEs) and any AE reported as an SAE will be summarised with the proportion of overall AEs and SAEs reported per randomised group. If appropriate, the proportion of subtypes of AEs and SAEs may be reported as well as the confidence interval and statistical test to estimate the difference between randomised groups.

**Table 1. Safety reporting responsibilities.**

| Type of Event | Who reports | To whom | When |
| --- | --- | --- | --- |
| All AEs and device deficiencies that occur at the site | PI assesses and records all AEs and device deficiencies | CPI, TCT, DSMC | Within 72 hours |
| All SAEs | PI | CPI | Within 24 hours |
| SSI instigated as USM | CPI | Reviewing HREC (SLHD – RPA)  DSMB  All investigators | Within 72 hours of notification |
| SSI not implemented as USM | CPI | Reviewing HREC  DSMC  All investigators | Within 15 days of notification |
| All SSIs | PI | The Research Governance officer for the site where the incident occurred. | As soon as possible and no later than 72 hours after PI aware of event. |
| USADEs | PI | The Research Governance officer for the site where the incident occurred. | Within 72 hours of the PI becoming aware of the event |
| Investigator Brochure updates | CPI | Reviewing HREC (SLHD – RPA) | When updates generated |
| Annual safety report | CPI | Reviewing HREC (SLHD – RPA) | Within annual progress report sent to HREC. |

*AE (Adverse Event) SAE (Serious Adverse Event) SSI (Significant Safety Issue) USM (Urgent Safety Measure) USADE (Unanticipated Serious Adverse Device Effect) PI (Principal Investigator) CPI (Coordinating Principal Investigator) DSMC Data Safety Management Committee) HREC ( Human Research Ethics Committee) SLHD (Sydney Local Health District)*

*Adapted from National Health and Medical Research Council (2016). Guidance: Safety monitoring and reporting in clinical trials involving therapeutic goods. Canberra: National Health and Medical Research Council.*

**Figure 1. Flow diagram of safety reporting responsibilities.**

*AE (Adverse Event) SAE (Serious Adverse Event) SSI (Significant Safety Issue) USM (Urgent Safety Measure) USADE (Unanticipated Serious Adverse Device Effect) PI (Principal Investigator) CPI (Coordinating Principal Investigator) DSMC Data Safety Management Committee) HREC ( Human Research Ethics Committee TGA (Therapeutic Goods Administration)*

*Adapted from National Health and Medical Research Council (2016). Guidance: Safety monitoring and reporting in clinical trials involving therapeutic goods. Canberra: National Health and Medical Research Council.*
